# Supplementary material for: Stakeholders perspective of integrating female genital schistosomiasis into HIV care: A qualitative study in Ghana
Source: PLoS Negl Trop Dis. 2025 Jun 2;19(6):e0012469. doi: 10.1371/journal.pntd.0012469 (PMC12169530; doi:10.1371/journal.pntd.0012469)
Supplement: S1 File — (DOCX) [file pntd.0012469.s001.docx]

**Stakeholders Perspectives of the Integration of Prevention and Control Measures for Female Genital Schistosomiasis (FGS) and HIV Care in Ghana**

**Key Informant Interview (Stakeholders)**

**IDI**

I: Greetings…

R: Response

I: How is your household faring?

R: They are going great. What of you please?

I: Great,

I: I would want us to talk about HIV and AIDS. Please tell me all you know about HIV and AIDS.

R: What I know about HIV and AIDS is that it is a deadly disease that is transferred from an infected person to another. It is transferred with infected sharp objects. In addition, transfer it through unprotected sex.

I: From where did you hear about HIV and AIDS?

R: I am have been an assembly member and a presiding member in the Ga South Municipality. We have been educated on these health issues. In addition, I got information from the radio and the TV.

I: How useful was the information?

R: It is very important. It helps us to protect ourselves. Ones you decide to have sex with anyone excluding your wife, you need to use condom to protect yourself. Also, avoid using sharp instrument such as blade with multiple people. The information is very important. Ones, infected, it can be transferred to other people.

I: Upon receiving this information concerning HIV and AIDS, Did it prompt you to take action about your health?

R: Yes. I took many measures to protect myself.

I: What action did that information made you to take about your health?

R: From the first day I received information on HIV and AIDS, I have protected myself. I do not use the same sharp objects with others. In addition, I am very careful whom I associate about sex. In addition, I make sure the gents' hair stylist sterilizes all instruments used when barbering my hair.

I: Would you have liked the information from any other different source?

R: Information on HIV and AIDS must be translated into all languages because Ghana is made of different tribes with different languages. This information must be spread at the market places and community centres. As I speak to you now, I am a member of health committee member. I would suggest that community health nurses visit the various markets to educate traders on the health situation we are talking about.

I: Do you know about any preventive measures?

R: Ones a person is born, with time you should be able to determine what is good for you and what is not good for you. Individuals have been trained on HIV and AIDS. All kinds of education provided by well trained professionals must be accepted and used the public. All preventive measure must be put in place to ensure we protect ourselves.

I: What preventive measures do you know for HIV and AIDS?

R: Avoid multiple use of sharp objects… also protect yourself when ones you engage in sex with multiple sex partners.

I: Since you have been an assembly and a presiding member before, I would like to find out from you, ones people are infect with HIV and AIDS, where do they seek care.

R: The visit the hospitals. Before HIV and AIDS is diagnosed, unless you visit the hospitals or clinics. Once HIV and AIDS is confirmed, the health workers take you through rudiments of treatment to manage the conditions to suppress the viral load.

I: Apart from the hospital, which other places do people seek care for HIV and AIDS?

R: To be honest with you, education on HIV and AIDS have enlightened many on the need to visit the hospital or health centre for HIV and AIDS care. To be honest, some also visit the herbal centres. Personally, I have never seen anyone who has been cured from HIV and AIDS with herbal medications.

I: What type of care is sought from each place?

R: They come for medications… this manage the condition until you die. In addition, they educate the clients on the HIV and AID. They also provide counseling services at the hospital. The HIV and AIDS patients are even told that they can give birth with such condition without infecting the babies.

I: What of the herbalist?

R: They prepare herbal concoction for the patients.

I: Please tell me about those who provide the care.

R: Those who provide care at hospital includes counselors, lab technicians and doctors. Nurse also provide care.

I: What of the herbal?

R: I can say much on the herbal. They are usually individuals.

I: In order of the most effective, list the most preferred place for care.

R: Many prefer the hospitals because medication For HIV and AIDS is free. In addition, patients are given monthly stipends to spend on their health and feeding since family members upon receiving information on the HIV and AIDS infection neglect some patients.

I: What do you think weaknesses and strengths of the care provided?

R: The strength of the hospital outweighs that of the weakness. The hospitals conduct a lot of screening freely for people to know their status. That is the first thing. Ones, a case is confirmed, special treatment is giving out to the person.

I: What of their weaknesses?

R: I have not observed any weakness. I am pleased with the approach used by care providers at the HIV and AIDS unit. They speak well.

I: In your opinion, what could have been done more effectively for each of the places mentioned?

R: The health workers need to be motivated. Especially, the community health nurses faces many challenges. One notable challenge is with transportation. They mode of transportation from the hospitals or clinics to the community must be relooked at and supported.

I: What can you about places that provide care using herbal medications?

R: They need to be integrated into a formal system. They need to be regulated. They must be hired and trained by the hospitals and Ghana health service.

I: I would want to ask you some questions about female genital schistosomiasis. Please tell me all you know about FGS.

R: This type of sickness can be cured at the hospital. Upon identifying the signs and symptoms, one need to quickly visit the hospital for treatment. Previously as an assembly member we advised community members not to swim in water bodies. The causative agent of FGS can be found in small rivers and ponds. As soon as you swim in a stagnant water, there is a possibility of an infection. When you visit most of the communities around the water bodies, most girls, boys, women, and even men are infected. For example, there are seasons where there is spillage from the Weija dam. The spilled water finds itself in homes, parks among other places. Individuals and households use these spilled water for bathing, cleaning and drinking. This becomes the source for infection. Immediately, you witness any sign of blood urination, you need to quickly visit the hospital for treatment. Delay in treating may results to several health conditions.

I: Please, where did you hear about FGS?

R: Weija. I have been an assembly member before. I spend most of my childhood days in Weija. My father was the former chief of Weija. Many of us swam in the Weija dam. People later complained of blood in urine. That was how I have to know about FGS. The moment you spend a lot of time in stagnant water, the possibility of infection is high. Krokrobite is another infection spot for FGS. They have a river that which many people derive their daily livelihood through fishing. Most of these people swim in the river and urinate as well while swimming. Most of these children swim in the river without any dress. Ones, you are naked and find yourself in the river, there is a high possibility of infection. Most of the villages in and around Amasaman have high cases of FGS. I have been an assembly member before as I said earlier. Most of these cases are reported at the health centres.

I: How useful was the information?

R: Very useful. It helps us to protect ourselves.

I: Did it prompt you to take action with regards to your health?

R: Yes. Personally, I do not joke with my health. I listened attentively to health workers. Personally, I do not usually take medications. I take in a lot of ginger, cloves, and garlic.

I: What action did that information made you to take with regards to your health?

R: I do not bath nor swim in these waters likewise my family. We currently have pipe borne water and boreholes.

I: Would you have liked the information from any other different source?

R: Yes, there must be community education on the FGS. In addition, the media should also take it up. I: Do you know about any preventive measures?

R: The first thing is to avoid bathing the river naked. Also, avoid urinating in the river nor pond.

I: Earlier, you mentioned that people with FGS seek care at the hospitals. Apart from the hospitals, which other places do people seek care for FGS?

R: Others also visit herbalist. Some herbalist have detailed knowledge on these conditions and what to do to combat the FGS. Most the trees, leaves, and roots among us can be used to treat many illnesses. Even banana peels and leaves are medications. As we are seated here, I have cloves soaked in water. I drink it every day. This cleanses your intestine. It also helps in metabolism.

I: What type of care is sought from each place?

R: Usually, the herbal give herbal medication. While the hospitals diagnose and gives medications.

I: Please tell me about those who provide the care

R: The hospitals have nurses and doctors while the herbalist are people who either inherited the knowledge or learned it.

I: In order of the most effective, between the herbal and the care provision at the hospital, which is the most preferred place for care?

R: The hospital.

I: Why?

R: Because of trust issues. The hospitals have specialist for treating these cases. That is why many prefer the herbal. Many visit the hospital.

I: What are the strength and the weaknesses of the care provision at the herbal centres?

R: Encourage people. However, some of the care providers are liars. The develop stories to make money.

I: What of the hospitals and health centres? What are the strength and weaknesses?

R: Proper diagnoses of health condition is the strength. On the other hand, some of the nurses exhibit negative attitude towards clients.

I: In your opinion, what could have been done more effectively for each of the places mentioned?

R: I suggest both the herbal providers and the clinical staff at the hospital to be educated. Research also needs to be undertaking. Health agencies need to integrate the voices of the masses into the health care provision.

I: Before we end our discussion, I would like to seek your opinion about integrating care provision for FGS, HIV, and AIDS in the community. Please tell me about your opinion regarding integrating FGS and HIV care.

R: It is not a bad idea. The HIV and AIDS has a department and FGS has its own department. The integration of the two conditions will not be difficult.

I: Would be acceptable to receive FGS care at the place where HIV care is provided?

R: Am not sure, it will be feasible. Per clinical knowledge, I think it will be appropriate to receive care of both HIV and AIDS and FGS at the same place. These are two different conditions and need different care.

I: would the community accept it?

R: It will not be accepted because of stigma attached to HIV and AIDS. There must be separation.

I: What do you think could be the barriers towards the integration?

R: There will be no barrier. The only barrier could be the long waiting time at the hospitals since one person will be providing care for both HIV and AIDS patients as well as FGS patients.

I: As an individual, what do you see as potential barriers to you going for an integrated provision of care for FGS and HIV?

R: There will be no barriers. There must be separation

I: At the community level, what do you see as potential barriers to the integration of FGS and HIV care?

R: I do not see any barrier. There must be separation of care since both conditions need different attention.

I: At the facility level, what do you see as potential barriers to the integration of FGS and HIV care?

R: Health workers fatigue since the same nurses and doctors need to attend to both conditions.

I: As an individual, what do you see as potential facilitators that will enable you to go for an integrated provision of care for FGS and HIV?

R: It will depend on the education. Individuals need to educate on the integration.

I: At the community level, what do you see as potential facilitators to the integration of FGS and HIV care?

R: They must organize a community durbar. The chiefs, the assembly members, unit committee members and the entire community must be educated on the integration. In addition, there will be the need to use the community health volunteers to educate the community. In addition, the public address system in the community must be utilized to send information on the integration across.

I: At the facility level, what do you see as potential facilitators to the integration of FGS and HIV care?

R: The nurses and doctors are available. All they need is logistics to work with. With sufficient logistics, the integration of care will be successful. Equipment is needed. The various institutions must resourced.

I: This bring us to the end of our discussion. Thank you very much for your time and responses. Please, do you have any question, contribution or subtraction from the discussion beginning until now?

R: I would want to express my appreciation for involving me in this discussion. Education on HIV and AIDS must be intensified. Over the years, education on HIV has subsided and needed to be intensified. FGS has been with the community for so many years without any education. Education on FGS need to carried out in the communities and the various schools in the municipality.

**Stakeholders Perspectives of the Integration of Prevention and Control Measures for Female Genital Schistosomiasis (FGS) and HIV Care in Ghana**

**Key Informant Interview (Stakeholders)**

**IDI**

**DEMOGRAPHIC DATA**

I: So, first, I want express my appreciation for your time.

R: You most welcome.

I: Please tell me all you know about HIV and AIDS.

R: The little I know about HIV and AIDs is that… it is sickness that attacks the immune system. It breaks down the immune system and gives way to several sicknesses and diseases. If the HIV virus is not dealt with, it will lead to AIDS. This may lead to death.

I: Please, where did you hear about HIV and AIDS?

R: I heard it from a church programme. We have a health programme in my church, which is organized yearly. Resources persons are invited to educate us on some of these health conditions. We also have some doctors and nurses in our church who educates us on some these health conditions. I am a church administrator

I: How useful was the information?

R: It has several importance. Before I continue, I want you to know that my biological mother died of HIV/AIDS. As a result, it has helped us a lot to know much about HIV/AIDS. The information helps one to protect him/herself from HIV/AIDS.

I: Did it prompt you to take action about your health?

I: Yes. As we speak now, I check my HIV status every three month. In addition, I check for high blood pressure as well as my sugar level.

I: What action did that information made you to take about your health?

R: I always take precaution on my health related issues. I constantly check my health status. Especially HIV/AIDS since it is free.

I: Would you have liked the information from any other different source?

R: Yes. I would prefer other sources. I wish to gain information from social media, television, and radio among other sources.

I: What other different sources will you like the information from?

R: House to house visit will also help just like the approach used by the community health personnel’s. They visit expectant mothers in their homes and they educate them on several health issues. This approach will help.

I: Do you know about any preventive measures?

R: We need to be careful.

I: How? What preventive measures do you know for HIV and AIDS?

R: First, it is not appropriate to share the same sharp instrument with people. For example, staying in the compound house. It is not appropriate to share items like tooth brush, towel, sharp objects… we need to make sure that sharp objects are sterilized at the barbering salon. You need to be careful.

I: With regards to care provision for HIV and AIDS, Please tell me about places where you seek care for HIV and AIDS.

R: Let me use my mother’s health issue as a case, when my mother was diagnosed of HIV, she taught that was the end of her life. Many people advised her to seek care from spiritualist and herbalist. Many people roam seeking for care. However, I think hospitals are the best place to seek for care. We advise our mother to seek care from the hospital. She refused. If she had listened to us, she would be alive today. It is better to seek for care at the hospitals or health centres. The government provides free medications for HIV and AIDS patients.

I: Not to remind you of the loss, however, please accept my condolences of the demise of your mother. To ask a little question concerning your mums health, where did she sought for care?

R: She visited spiritual centres and religious organizations. She also visited herbalist. She came home with concoctions in gallons.

I: *What type of care is sought from each place?*

R: Incantations, chanting, prayers, among others are the types of care sough from spiritual centres. For example, my mum was giving some concoctions to drink and to bath with. In addition, she was made to perform some rituals.

I: What of the hospitals, what type of care is sought form the hospitals?

R: Well… hospitals are the best places to visit. I what I know is that the health workers are well trained. They diagnose and manage the conditions. I will call for support for the hospitals. Recently, we heard that the hospitals run out of HIV and AIDS medications. Frequent provision to these medications will help.

I: Please tell me about those who provide the care.

R: at the hospitals, trained personnel’s provide care while the spiritualist are individuals who probably had knowledge from ancestors.

I: In order of the most effective, list the most preferred place for care.

R: In the olden days, many people sought spiritual guidance from difference sources. It can be said today. I will say that many people prefer the spiritualist as compared to the hospitals.

I: Why the preferences?

R: Many people say that they are cured when they visit the spiritualist. I personally, I will prefer the hospital. I will rather take instructions from health personnel’s than spiritualist.

I: What are the strength and the weaknesses of the spiritualist?

R: Many of the spiritualist have not been trained and certified to provide care. Most of the them know them that this medication will be good for the patient but they do not take into consideration the blood type of the patient among other vital issues to consider before giving out medications. Most of the them do not have license to operate. Also, environmental conditions for preparing of their medications are unclean.

I: What of their strength?

R: I personally cannot talk about their strength due to experiences, which caused the death of my mother.

I: What of the hospitals? What are their strength and weaknesses?

R: The only weakness I can say about the health practitioners is that most of them do not have the patience to manage clients. They are often rude to clients. On the other hand, they are well trained and that is the strength. They the know the medications for each conditions. They are also licensed.

I: In your opinion, what could have been done more effectively for each of the places mentioned?

R: They should continue training professionals. Also, the provision of medications and equipment to work in the hospitals.

I: What of the spiritualist and the herbalists?

R: Some herbalist have been certified. I will call for the FDA and Ghana Health Service to regulate their activities and find ways of merging both formal and informal institutions.

I: Okay. Thank you… we will now talk about female genitals schistosomiasis (FGS). I will want to find out about your knowledge on FGS. Please tell me all you know about FGS

R: I have never heard of female genital schistosomiasis. However, I have heard of female genital problems. Last month, some nurses came to the community to educate the women. I heard those mentioning FGS. They advised her on how she should take care of herself. Afterwards I went out to ask the nurses what FGS was. That was my first time of hearing female genital schistosomiasis. She advise me on how to manage such conditions. My children are all girls.

I: What did they say about FGS?

R: They said… FGS is an infection that affects the bladder. I was told to monitor her during her menstruation period. I was also informed that they should avoid swimming in stagnant water bodies. They said, they could be infected.

I: Upon hearing about this information, what did you do?

R: I advised my daughters to avoid swimming in water bodies.

I: Apart from the nurses, where other sources did you hear about FGS? The nurses came to the community to educate the women on the FGS.

I: Would you have liked the information from any other different source?

R: As I said earlier, the media and the social media since most people get information from social media. Instead focusing on politics, I suggest they focus on some of these health conditions.

I: Do you know about any preventive measures*?*

*R: What I know is that women need to take care of the genitals. They should avoid the use of duchess and locally prepared concoctions but rather, they should visit the hospital.*

*I:* Please tell me about places where you seek care for FGS.

R: I do not have much information on the FGS. However, I think the hospital would be the best place to seek for care.

I: *What type of care is sought from each place?*

R: FGS care… go through the process from diagnose to treatment. It is the work of gynecologist to treat this condition.

I: Why do you think the hospital is the best place to seek FGS care?

R: The workers at the hospital are trained and well equipped to provide care for FGS. That is why it is the best place.

I: What do you think are the *weaknesses and strengths of the care provided at the hospitals?*

*R: I can say much… What I can say is that the hospitals are the best place to provide FGS care.*

*I:* In your opinion, what could have been done more effectively for each of the places mentioned?

R: The government should resource those to enable them perform well.

I: Let us talk about integrating care provision for FGS, HIV, and AIDS in the community. Please tell me about your opinion regarding integrating FGS and HIV care.

R: In general, the main issue will be with the HIV and AIDS. HIV and AIDS care received more attention as compared to the FGS. I will call for equal attention. Nurses and doctors must visit the various communities and schools and educate the community members on HIV and AIDS. Health education must be intensified on these issues. This will reduce the rate of infection.

I: W*ould it be acceptable to receive FGS care at the place where HIV care is provided?*

*R: It depends on the health professionals and the way they will handle the situation. If clients are not informed about the status of each other, that will be fine. Nevertheless, if they are aware of the status HIV and AIDS clients, there will be many challenges, as many people do not want to associate with HIV and AIDS patients.*

*I: Do you think it will be acceptable?*

*R: Yes, it will be acceptable if the status of HIV and AIDS clients are unknown. In addition, FGS only affects women. What of the male HIV clients? That will be a challenge.*

*I:* As an individual, what do you see as potential barriers to you going for an integrated provision of care for FGS and HIV?

R: The only barrier will be the attitude of the health workers towards patients. Also, the separation of HIV and AIDS and FGS patients. This will lead to stigmatization. This will not help. However, if clients visit the hospital without any form of segregation, it will help reduce the stigma associated with HIV and AIDS.

I: Any other perceived barriers?

R: No! This is what I can say.

I: Also, at the community level, what do you see as potential barriers to the integration of FGS and HIV care?

R: Stigmatization associated with HIV and AIDS.

I: At the facility level, what do you see as potential barriers to the integration of FGS and HIV care?

R: Bad attitude of nurses especially.

I: On this note, I would want to find out from you the perceived implementation facilitators/enablers. So, as an individual, what do you see as potential facilitators that will enable you to go for an integrated provision of care for FGS and HIV?

R: Education. Individuals must be educated. They is not much information on FGS. Individuals need education. Likewise, education on HIV and AIDS has also reduced. People need to be sensitized on the integrations. People must be loved.

I: At the community level, what do you see as potential facilitators to the integration of FGS and HIV care?

R: Community education is the way to go. I would have preferred community health nurses to visit the various homes and educate them. Women staying beside water bodies should avoid bathing in them and treat the water bodies before using it.

I: At the facility level, what do you see as potential facilitators to the integration of FGS and HIV care?

R: The health workers need to be trained on the integration. In addition, there must be counselors at every health facility to counsel clients on the integration. This will help.

I: I would want to thank you for your responses and your time. Do you have any question, addition, or subtraction?

R: Logistics need to be provided to the various health facilities to enable them combat HIV and AIDS as well as FGS.

**Stakeholders Perspectives of the Integration of Prevention and Control Measures for Female Genital Schistosomiasis (FGS) and HIV Care in Ghana**

**In-Depth Interview (Community Health Committee Member)**

**IDI**

**DEMOGRAPHIC DATA**

**FACILITATOR/ INTERVIEWER:**

I: Interviewer

R: Respondent

**INTRODUCTION**

Let’s go ahead and get started. My name is and this is my colleague. Today we would like to have a conversation with you all. We are very interested in learning about integration of Female Genital Schistosomiasis into HIV care. We will only share the information we learn today in a general way that does not reveal your identity. With your permission, I would be recording our conversation. It’s important that the information shared does not leave this group. So we ask everyone to not share who was here or what was said with others outside when you leave here. We really want to hear what you have to say and want you to feel comfortable in answering questions however you want to. There are no right or wrong answers.

NII is taking notes to make sure that we don’t miss what you have to stay. This will help us later when we go back and organize all the information that was shared today. This discussion should last for about 30 minutes.

Do you have any questions before we begin?

***Ice Breaker***

Ask participants to wish for something good for their community in this year 2024. Ask why they want that particular wish for the community.

**I:** Thank you for your time for this discussion.

**R:** Please it is fine, Thank you.

1. **Knowledge about HIV and AIDS**
2. Please tell me all you know about HIV and AIDS.

**I:** I’ll like to know your opinion on what you know about HIV and AIDS.

**R:** With HIV and AIDS, it’s not just Males and Females that can contract HIV and AIDS, for instance if an individual maybe living with HIV and AIDS uses a blade to trim his/her nails and you use the same blade, you can get infected with the HIV and AIDS or a needle or an object, if someone has AIDS for a long time and you use that same object, you might get infected with AIDS. That is what I know about HIV/AIDS.

**I:** Please do you have anything else you’ll like to add to what you have said?

**R:** This is all I have to add.

1. From where did you hear about HIV and AIDS? *(****Probe*** *to know about all sources of information)*

**I**: Okay. Thank you. So please these things that you know about HIV, where did you hear about them?

**R:** Normally through announcements and also, excuse me, I’m not a Child so I know about HIV and AIDS for a long time. (Acquired Immune Deficiency Syndrome) so I know about HIV.

**I:** Aside the Announcements and what you know about HIV, have you heard about HIV and AIDS anywhere else?

**R:** I know some people from my town about four to five of them that were infected with HIV but they have been placed on HIV medication so now, they are normal and because of the medication, you wouldn’t know they have HIV and AIDS unless someone tells you.

1. How useful was the information?

**I:** So these information that you heard, do you think it was a useful information?

**R:** Yes why not? It is a very useful information and we have to follow it seriously

**I:** So How Useful was it?

**R:** With the Usefulness, its fine because if the thing is not there we wouldn’t talk about it, so it’s very useful we have to be very serious on that. It’s very useful.

1. Did it prompt you to take action with regards to your health? What action did that information made you to take with regards to your health?

**I:** What did you use the information you heard to protect yourself in terms of your health?

**R:** I told my children to be careful about the Wanzam people especially the Northerners and Hausa people who carry razor blades to trim nails of people. It’s very dangerous because when they trim the nails of people and it cuts them and because they don’t sterilize the knife they hold, so if they use it on you and it cuts you, you can get infected with HIV and AIDS. I tell them that when someone uses a blade to trim their nails, they shouldn’t use the same blade. They should buy their own blades, if it is a needle or any other thing, anything they would like to do, they should use their own things maybe that will be fine. And with Brushes, maybe you trust a friend so if you travel somewhere and he/she uses the brush and you use the same brush, you can sometimes get infected.

**I:** How this you use this information to protect yourself?

**R:** Because I know this myself, I am always very careful on that

1. Would you have liked the information from any other different source*?* ***(Probe*** *to know about which sources).* What other different sources will you like the information from?

**I:** You said you heard about HIV and AIDS through Announcements and what you read on your own right?

**R:** Yes, I read about it in newspapers because I buy newspapers always so I know and also on TV.

**I:** You didn’t mention these sources earlier, so we will like to know all the sources where you hear about HIV and AIDS.

**R:** Okay

**I:** Apart from the Sources you mentioned, do you have any other source that you wished you should’ve heard about HIV and AIDS?

**R:** What I know is what I have told you, but if you know any other source that you can tell me, I will be very happy.

**I:** This is a highly opinionated questions so I can’t express my view on this.

**R:** What I now is what I have told you.

**I:** Maybe is there a place in your community or at home or anywhere you feel you should have heard about HIV and AIDS that you haven’t heard?

**R:** Oh no

1. Do you know about any preventive measures*? (****Probe*** *to know what preventive measures.)* What preventive measures do you know for HIV and AIDS?

**I:** Do you know the way we prevent HIV and AIDS?

**R:** With the prevention of AIDS, Maybe if you have a girlfriend and you don’t trust her, you can use a condom and that’s what I know. So you can use a condom but apart from the condom use, what I said about the prevention you will have to think about that. As for Male and Females, you will have to use a condom. Even with the condoms, at times it is not safe because the condom can break during sexual intercourse and if your partner has the disease, you can get infected.

**I:** Please do you know any preventive measures aside the condom use and what you said earlier?

**R:** Apart from all I said earlier, this is all I have to say.

**I:** Please do you know about any other preventive measures?

**R:** With the other measures, that’s all I said earlier, but in the Northern Region which is our side, the females that do the Female Genital Mutilation, they don’t sterilize the knife they use which they are supposed to do. So sometimes people do contract HIV/AIDS through that.

**I:** So how can that be a preventive measure?

**R:** Ministry of Health and We (Health) need to educate them on that. It’s even not proper because the Government has placed a Ban on such activities so if someone does that, it’s illegal so they even hide to do it.

1. **Knowledge about FGS**
2. Please tell me all you know about FGS

**I:** I’ll like to find out your opinion on FGS. So tell me what you know about FGS.

**R:** Okay, FGS is a Disease which is not like HIV, but affects the private parts of women. They feel pains when they have sexual intercourse with their husbands, they also feel pains around their stomach area.

1. From where did you hear about FGS? *(****Probe*** *to know about all sources of information)*

**I:** From where did you hear about FGS?

**R:** From a friend. I didn’t hear about it like I heard about HIV but I was with my friend and he told me that people can get infected with this type of disease.

1. How useful was the information?

**I:** What you heard about FGS, do you see it to be very useful?

**R:** Yes it is Very useful but based on what my friend told me, it’s not like HIV

1. Did it prompt you to take action with regards to your health? What action did that information made you to take with regards to your health?

**I:** So the information you heard on FGS, how did it prompt you to protect yourself?

**R:** Okay, so what I have to say is that, if you have one girlfriend, stick to her. If you are a boy or a girl and you have multiple partners, you can be infected with this disease. But if you stay with only your partner, it will be difficult for you to get it.

**I:** Personally, what did you use the information for?

**R:** I used it to educate my children because I have 4 girls. Sometimes I talk to them about HIV and these diseases.

1. Would you have liked the information from any other different source*?* ***(Probe*** *to know about which sources).* What other different sources will you like the information from?

**I:** You said you heard about FGS from your friend, so apart from that friend, where else did you hear about it?

**R:** No, I haven’t heard about it anywhere else.

**I:** Do you have a place that you wished you heard more about FGS that you didn’t hear?

**R:** Truthfully a friend told me about this so I didn’t hear about it anywhere else like HIV/AIDS.

**I:** But don’t you have any place in mind where you wished you heard about FGS which you didn’t? And not to wait for your friend to tell you what FGS is?

**R:** Okay, so like what I said a friend told me, maybe the Hospitals because one of my daughters is a Nurse so I should be able to ask her. Or I could go to the facilities because I know people who could educate me more on this Disease.

1. Do you know about any preventive measures*? (****Probe*** *to know what preventive measures).* What preventive measures do you know for FGS?

**I:** Okay, So do you know how we can prevent FGS?

**R:** So like what I said in the beginning, you should have a stable partner. But if you have multiple partners, you can get infected with what I said.

1. **Opinion about care provision for HIV and AIDS**
2. Please tell me about places where you seek care for HIV and AIDS.

- ***Probe*** *to know if care is sought from multiple places.*
- ***Probe*** *to know what type of care is sought from each place.*

**I:** In your community if someone has HIV /AIDS where do they go for care and treatment?

**R:** If you go to the Hospital, no one will know that you are HIV positive and even with the FGS.

**I:** Right Now, we are talking about HIV

**R:** So if you go to the Hospital, that’s when they will see to you. Sometimes an individual might unknowingly be a carrier and will be carrying it around but will be detected when they get to the hospital.

**I:** So if they diagnose someone for HIV, where will the person go for treatment?

**R:** Hospital, The person will have to go to the hospital

**I:** Aside the Hospital, do you know of any other place that they go to seek for care?

**R:** Well, if you get infected with HIV, some people go to the Herbalist. People go to the Herbal Centers for treatment but to get healing, I’ve never heard of that. They go round and round and still end up at the Hospital.

**I:** Aside the Hospital and the Herbal Centers have you heard of any other place that infected people seek for care and treatment?

**R:** Some People also have their hopes on churches that maybe through prayers they might be healed so people do go to these places.

**I:** So if they Hospital, what kind of care or treatment do they go for?

**R:** When they go to the Hospital, they give them HIV treatment tablets and they go for them maybe monthly. If it gets finished and they go back, they give them more.

**I:** What of the Herbal Centers, what kind of care is being given there?

**R:** With that, they give them Herbal drugs which I know they pay for. But they said with the Government Hospitals if you go, they don’t take money or something like that.

**I:** So those that go to the churches what type of care do they give to them?

**R:** They don’t receive any care, all they give them is prayers believing that they will be healed.

1. Please tell me about those who provide the care

**I:** So at the Hospitals, who provides the care?

**R:** The Nurses and the Doctors. You’ll go to see the doctor to prescribe the drugs then you go for it.

**I:** And at the Herbal Centres, who provides the care?

**R:** the Herbal Doctors

**I:** And at the churches who provides the care?

**R:** The Pastors. They will pray for you.

1. In order of the most effective, list the most preferred place for care *(****Probe*** *on why the places were ranked in a certain way, weaknesses and strengths of the care provided)*
2. In your opinion, what could have been done more effectively for each of the places mentioned?

**I:** You mentioned Hospital, Herbalist and Churches, I will like you to rank them. So which one will you place first?

**R:** I will place Hospital 1st, Herbal Centers 2nd and Church 3rd.

**I:** What is the reason for ranking the Hospital first?

**R**: I know when they go to the Hospital and they receive the HIV medications, they recover that’s why I ranked the hospital first.

**I:** Why did you rank the herbal centres second?

**R:** With the herbal, because it’s HIV/AIDS, they have very powerful medicines that can reduce the HIV AIDS virus in the system

**I:** Why did you rank the church last?

**R:** With the church, it can work but the reason I ranked them last is that, some pastors can be sneaky, they just want to take money from you and they will do the prayers but I’m not saying all Pastors are like that but the genuine ones are there who will pray for you. I ranked the church last because of the fake pastors.

**I:** At the Hospital, what do you think they don’t do well when treating people living with HIV/AIDS? Looking at their strength and their weakness.

**R:** In the Hospital, when they take the HIV drugs, they get the Strength. First, they will be slim but when they start with the medication they recover. So when you see such an individual, you wouldn’t know they have the virus because of the drugs they receive at the hospital.

**I:** So the Hospital Itself, what care do they give to the clients that makes them happy?

**R:** I have not had some before so if they visit the hospital, I am not there to see what goes on.

**I:** We just want to get your view on this issue

**R:** Okay so the hospital, they treat them well, and if they go, they see to them fine

**I:** And what do you think they don’t do well at the Hospital?

**R:** I don’t have anything negative to say because I know when they see to them at the hospital, they don’t take money from them.

**I:** can you share with me the positives and negatives of the Herbal Centres?

**R:** With the positive side, I said it earlier that they have herbal drugs that are very powerful. The bad side is that, the money they take from these clients are very extravagant which they have to do something about because since the Government doesn’t charge they can do something about the money they take because it’s too much.

**I:** What about the positives and negatives of the Church?

**R:** The positive side is that they will pray for you and the negative is that they will charge a fee. If you are helping someone with prayers, you don’t have to take money from that person but they do take money from them. But because of the money they charge, I consider that to be a negative thing.

1. Apart from the places mentioned, is there any other place you would have preferred to go for health care? (***Probe*** *on the reason why*)

**I:** Aside the places that you have mentioned, do you have any other place in mind?

**R:** No

1. **Opinion about care provision for FGS**
2. Please tell me about places where you seek care for FGS.

- ***Probe*** *to know if care is sought from multiple places.*
- ***Probe*** *to know what type of care is sought from each place.*

**I:** In your community, if someone has been diagnosed of FGS where do they go to seek treatment and care

**R:** They go to the Hospital.

**I:** Do you know of any other place aside the hospital?

**R:** From the beginning when they get infected, they sometimes go to the Herbal centres where they receive herbal drugs but the finally go to the Hospital.

**I:** You mentioned Herbal Centres and Hospitals. Do you have any other source to add?

**R:** No. I don’t know of any other source.

**I:** What type of care do they receive when they get to the Hospital?

**R:** When the visit the hospital, they go to see the doctor who will request for lab testing and results through which they will be diagnosed. After these the Doctor will write prescription for them to go and purchase.

**I:** And what of the Herbal Centre, what kind of care do they receive there?

**R:** Some of the herbal centres have a lab that they test. But some don’t have so when you with symptoms like stomach ache and private part complications, they just give you herbal medicines without testing. That is what most of them do.

1. Please tell me about those who provide the care

**I:**  Who provides the care at the Hospital?

**R**: Doctors and Nurses

**I:** Who provides care at the Herbal centre?

**R:** The Herbal Doctor and their Nurse

1. In order of the most effective, list the most preferred place for care *(****Probe*** *on why the places were ranked in a certain way, weaknesses and strengths of the care provided)*

**I:** Between the Hospital and the Herbal Centre rank them for me.

**R:** I would rank the Hospital first because they take your blood for testing and the drugs they will give you are very effective and cure them early.

**I:** So why did you rank the Herbal Centre second?

**R:** like I said earlier, some of the Herbal Centres are good that’s why I placed them second.

**I:** I will like to find out the Positive and Negative sides of the Hospital in terms of the quality of care

**R:** When you visit the hospital, the doctors have the time to take care of you, they will request for lab and when you are diagnosed with the illness, they will give you drugs which makes you recover in no time. I don’t have anything negative to say about the Hospital

**I:** What about the positives and negatives of the Herbal Centres?

**R:** the positive is that, they give you medicine for treatment and the negative side is that they sometimes give out medicines they know will not be effective just to take money from you. Some of them when you tell them you’ll like to go to the hospital, they won’t allow you to telling you that the drugs will work. But when you will get to realize that you are wasting your time with them, you go back to the Hospital.

1. In your opinion, what could have been done more effectively for each of the places mentioned?

**I:** In your opinion, what do you think can be done more effectively of the two sources you mentioned?

**R:** the Hospital is the best, because when you get infected with any illness, the hospital should be your first resort.

**I:** So what are they not doing well that you feel they should do well?

**R:** let me talk first about the hospital. When you are Ill and you visit the Hospital, the way some of the nurses talk to you sometimes isn’t good because when someone is ill you have to talk to them calmly. But some of the nurses don’t talk well to the clients that’s what I see to be negative

1. **Opinion about integrating care provision for FGS and HIV and AIDS in the community**

Please tell me about your opinion regarding integrating FGS and HIV care.

- ***Probe*** *to know if it would be acceptable to receive FGS care at the place where HIV care is provided.*
- ***Probe*** *to know reasons why it would be acceptable or unacceptable.*

**I:** Please tell me about your opinion on the integration of FGS and HIV care. What do you have to say about the Integration?

**R:** The integration wouldn’t be effective if they want to separate the care for both conditions. But if they integrate it, it will be fine.

**I:** In the Facility, there is a place for HIV care, do you think it will be acceptable if they ask those with the FGS condition to go to that same unit for care?

**R:** Yes they will accept it simply because when you eat or shake hands with HIV patients, you won’t be affected. Because when you go to the same unit for care they wouldn’t use blades there to cut you or a needle to inject you. So I think if they merge it, it will be very effective and fine

**I:** let us assume you are a lady with FGS, when you visit the Hospital for care and you are being directed to the HIV to receive treatment, would you accept it?

**R:** [Laughs] Yes I would accept it because if they use a needle in the unit, they discard it and use a new needle if the need be. So if they integrate the care for both, nothing will happen to any individual.

1. **Perceived implementation barriers**
2. As an individual, what do you see as potential barriers to you going for an integrated provision of care for FGS and HIV?

**I**: So as an individual, being it yourself, your wife or your child, what do you think will prevent the integration from being effective?

**R**: To me for the Integration not to go well, I don’t see anything that will prevent it from going well because by all means it will go well. Referring back to what I said about the use of the needles in the unit, it will surely go on well that is what I will like to say because it’s okay

**I:** We are going on with the discussion, what challenge do you think will be encountered in the process of the Integration?

**R:** like I said earlier, I don’t think it will come with any problem, probably if they come together, it won’t be fine if they ask HIV patients to sit somewhere and FGS patients to also sit somewhere else. So they should all be together.

**I:** Since you want them to be integrated, what challenge do you foresee?

**R:** According to me, I don’t think there will be any problem encountered.

1. At the community level, what do you see as potential barriers to the integration of FGS and HIV care?

**I:** Ok, then what challenge do you think will be faced relating to the community?

**R:** Sometimes the community will easily identify the people with HIV and the FGS if they ask them to sit separately as HIV and the others. The separation will be a problem but if they allow them mingle together as one, there will be no problem

1. At the facility level, what do you see as potential barriers to the integration of FGS and HIV care?

**I:** So at the health center, what challenges do you think will be faced in trying to integrate the care for FGS and HIV/AIDS?

**R:** I don’t think it will come with any problem. But if they allow them to sit separately as HIV patients and FGS patients, people will get to know their condition and this will bring problems.

1. **Perceived implementation facilitators/enablers**
2. As an individual, what do you see as potential facilitators that will enable you to go for an integrated provision of care for FGS and HIV?
3. At the community level, what do you see as potential facilitators to the integration of FGS and HIV care?

**I:** So what will be the challenge that you raised, what do you think will enable or make the integration successful.

**R:** I think there should be more announcement to inform the public and the individuals that we are integrating the care for FGS with the care of HIV/AIDS. I think when they report together, there won’t be any problem because there will be separate medication for them.

1. At the facility level, what do you see as potential facilitators to the integration of FGS and HIV care?

**I:** So what about the Hospital, what do you think will enable the integration to be successful?

**R:** I will repeat myself that if the treatment is integrated, there will be no problem because I understand there is no seperate injection for HIV/AIDS and there is no injection for FGS as well so if the treatment will be integrated, there won’t be any problem.

**I:** Thank You for your time.

**R:** Welcome and thank you too.

**IDI CHCM 4**

INTERVIEW WITH HEALTH COMMITTEE MEMBER

KEY WORDS:

I: INTERVIEWER

R: RESPONDENT

I: good morning

R: good morning

I: my name is Angela from the school of public health, and we are conducting a study on the integration of care for HIV and FGS patients, and as a health committee member we would like to seek your view concerning this integration, the barriers as well as it's enabling factors that will help with the integration purpose, I also assure you of confidentiality, and that this discussion will be used solely for the integration purpose only. Before we start with the interview, I would like to get some bio data from you, how old are you please?

R: 70 years

I: your marital status?

R: I'm a widow

I: what is your highest level of education?

R: form four

I: your religion?

R: I'm a Christian

I: what do you do for a living?

R: I'm a trader

I: what tribe are you?

R: I'm a fanti

I: how many children do you have?

R: 6, 4 are my biological children and 2 are adopted children

I: where do you stay?

R: amanfrom

I: okay, please, what do you know about HIV?

R: I know HIV is caused by a virus, initially people had the perception that it was gotten through coming into contact with some animals like mouse but research has shown done it's caused by a virus through coming into contact with an infected person, either by having sex with an infected person or using sharp objects with an infected person, you can only get it if you come into contact with an infected blood, so it's caused by a virus, that is what I knew about it initially

I: okay, so, what else do you know about it now?

R:now, I've heard you can be put on medication and you can live a normal and a healthy life once you adhere to the medications unlike initially where people die and grow lean, though the medicine can't cure you, it helps you live longer and have a normal life, just some few months back, there is this lady I know whose daughter was diagnosed of having HIV, it hasn't developed into AIDS yet, and she's on medication, I didn't believe her initially because the girl looks healthy and strong and doesn't look sick, so now I know with medication you can have it and still look healthy and good unlike initially where they looked sick and grew very lean, so, I though probably the rate of infection was low now but I heard it's rather on the rise so it's the medication that is helping them and you don't see them sick and growing lean anymore

I: okay, so where did you get to hear of all this information about HIV from?

R: from the news and through research on my smart phone, and also my sister is diabetic so I users to accompany her to korlebu for checkup and her medication, so when I go and they are educating people about health issues and HIV, I go and listen, so that I can also educate others about it because it's a very serious sickness, so I got the information from the news, my smart phone as well from korlebu

I: okay, so which media channel did you hear the news on?

R: I normally listen to peace FM, so I heard it from peace FM and then the television

I: okay, so how the information received been useful to you?

R: I'm able to educate the young ones about the disease and even with my own children, I educate them, when my second born wanted to get married, I advised her to go together with her partner to go and have an HIV test done so they know their status because that is what I've heard, so she they should go and have it done, because one might have it and you wouldn't even know until they get tested, so they went to have the test done, so it has helped me because probably the man might have it and my daughter too would have been infected so that it doesn't bring problems to the family, so for me, once you are closer to me I will educate you about HIV

I: okay, but personally, how has the information about HIV helped you?

R: I'm very cautious of my health now, I have hypertension but my mother is old now and she's frustrating and worrying me now, so I keep thinking about it and that is what is making my blood pressure go up, but 2 of my children are nurse and one is a lab technician at korlebu so I frequently go for tests, at least after every 8 months I go for a lab test so it has helped me to be vigilant and be cautious of my health

I: okay, what preventive measures do you know about HIV?

R: as I said earlier, aside being medication when you get it, you need to protect yourself also by avoiding having sex with multiple partners and not sharing sharp objects like blade with people, and then you should avoid coming into contact with blood, I know HIV is all about coming into contact with an infected blood so try as much as possible to stay away from blood and fresh wounds or people who have wound, even if you want to touch the wound you need to protect yourself

I: are there any other sources of information you would like to get information about HIV from?

R: yes, I think the other sources like how you have come here and the youth who are done with their national service and not working now should go round to talk to people about it, because there are people who still don't believe HIV is real just like when covid came, someone told me that it's a means for the government to make money so there's no covid, but I personally had covid, so the education and sensitization is key, at least some of the youth can be sent on outreach to educate people on HIV during national service, because most of the youth don't believe HIV is real and the medication is expensive and catered for by the government, my community here, most of the ladies here are just living promiscuous lives, sleeping around and doing all sort of things so they should be outreach to educate the young ladies about it

I: okay, is it only the young ladies who needs to be educated about HIV?

R: no, not only the females, but everyone both young and old, they should be duly educated so that they can also educate others

I: so they should be more outreaches which we can make use of the national service personnel?

R: yes, because I think the education is still not enough, all though it is financial draining but it's for the best because when one person contracts the disease, he or she can spread it to a lot of people without knowing that's why I'm making this suggestion

I: okay, what other sources again will be helpful?

R: in this community, for example, they do weighing of babies in my house so sometimes they give health talks, so I call some of the youth to come and listen and tell them to adhere to the preventive measures, some of the lactating mothers don't even bring their infants for weighing and immunization so when I see them, I advise them to do so, and then we don't have to wait on government to do everything for us, as a community we can educate each other but the problem is everybody is looking for money before they will agree to get involved in anything, but the nurses here are doing well, once in a while they organize health talks for the family but mostly are about lactating mothers and babies, so probably just as you've come like this, a day can be set aside for a durbar and we talk to them about HIV/AIDS, it will help, the chief's, opinion leaders and other authorities can get involved to help with the education and sensitization

I: okay, what do you know also about FGS?

R: I've heard of it before but I didn't know it was still in existence, it was 3 days ago that I was asked to come to the hospital and the nurse told me those who live by the river still contract it and they come to the hospital for treatment, it was when we were kids and go and bath in the river that we used to have it, and people will be urinating blood and all that but I didn't know it was still in existence so I was surprised when the nurse told me about it

I: okay, so what do you know about it?

R: I know that if you don't prepare your water well after fetching from the river and you use it you can have it or you normally take your bath in the river, there are some living organisms in the water that causes FGS, that’s what I know but I don't really know how it's like

I: okay, so where did you get this information from?

R: when I was a kid, I was at obuase and there was a river on our way to the farm where we go and swim and bath inside, people used to complain of urinating blood after bathing in the water but personally I never experienced it before

I: so how did the information you had about FGS while growing helped you?

R: we stopped taking our bath in the water and my mother used to boil our water before drinking it so I never had it

I: okay, as you said earlier, you heard about it a long time ago

R: yes

I: so how can we educate people about it now?

R: it is also caused by a virus too so when they educating the public about HIV, they can add it as well

I: so, what about the radio and television you mentioned earlier for HIV?

R: that one too will be very good, I'm even surprised it's still existing so that especially those by the river sides should be probably educated because they mostly attribute such things to spirituality so it will be good to educate the public

I: where do people go to for treatment in your community when they have HIV?

R: honestly I've lived here for 25 years but I don't know of anyone personally who have HIV so I don't know where they go to for treatment, but I know they have a unit at korlebu where they attend to them and take their medications

I: okay, as a committee health member, where do you think people go for treatment in this community when they have HIV?

R: we tell them to go to the hospital when they are sick, and even if there's no doctor to attend to you, you will be referred to a bigger hospital so they come to the hospital

I: do your community members come to the hospital when they are sick?

R: yes they do, now we even have a PA here so he will attend to you, initially we used to go to kasoa Poku clinic but now we come here

I: aside the hospital, which other places do you think they patronize for treatment for HIV, maybe the herbal centers?

R: as for that one I can't tell, it's just that most of the people here are ewes so sometimes they travel to their home town when they are sick but I don't know what they go to do there, but for me once I'm closer to you, I will advise you to go to the hospital first before any other place for treatment but I don't know where they do and go privately

I: what do you think about the care you receive from the hospital?

R: it’s okay, it's the knowledge the doctors have that they use in treating us, so if you adhere to whatever you are being told and take your medications well, you will be fine,

I: okay, but what do you think can be done to improve on health care for HIV patients by the health centers?

R: I think the health workers should be doing a regular follow up to check whether they are taking their medications as prescribed

I: do you think your community members would like to go to the hospital when they get FGS?

R: yes, they will, because all of a sudden you start urinating blood, and you don't know it's cause so you have to go, there is this lady I know, recently she started bleeding from the nose, I asked what was wrong with her and she said she doesn't know so I told her to go to the hospital, she said she had no money so I had to go and borrow money to give to her so she can go to the hospital for treatment, when she went to do a scan at the hospital, it was diagnosed that it was her liver that was causing the bleeding, she had typhoid and wasn't treated well and it has now affected her liver, and she drinks too so she was advised by the doctor to quit drinking alcohol and was given medication and now she's okay, so when you see somebody with a condition you need to advice the person to go to the hospital, don't say probably because he or she is not your family member so you don't care

I: okay, so now let's look at the integration of care for HIV and FGS patients, what is your opinion on the integration?

R: I see nothing wrong with it because they are all infection, if you have HIV it's an infection likewise FGS, nobody will use any sharp object like knife or blade here for you to say you will get infected, unless the person has flu, because for flu if the fluid of the person comes into contact with you through coughing or talking you can get it but with HIV and FGS, it's not like that, you can talk with the person, sit together and you won't have it so I see nothing wrong with it

I: okay, do you think it will be accepted by the community?

R: it will depend on the education and sensitization, if they are educated well, they will accept it, and they need to be educated that HIV is not like covid that you can get by coining close to the person

I: what do you think will be a barrier to the community not to accept the integration?

R: we have some people that won't accept change no matter what, but as I said earlier, if the education and sensitization should be done well I think they will accept it

I: as a community health member, what do you think will be a barrier to you accepting this integration?

R: me, personally I don't think they will be any barrier,

I: at the facility level, what do you think will be a barrier to the implementation of this integration?

R: I will use this hospital as an example, when people come to help the hospital, instead of them to allow them finish or complete the hospital, they will allow the person to go and start a new building altogether, when I came here and I saw the new maternity ward I was impressed, so if the facility is spacious enough where patients who come will feel comfortable it will be okay but if they come and they don't get a place to sit and they are just seated under the tree, then the will be a challenge and if they come and they don't meet a doctor too it will be a problem

I: okay, so infrastructure

R: yes, when you come and maybe I have to go and do lab test elsewhere then it will be a problem, so if I sick and I know when I come, they will demand I do a lab test I won't come here at all, I will go to kasoa, so if the hospital has everything, there is lab, medication and doctors available they will come and there wouldn't be any barrier, initially when you tell people to go to the hospital, they will tell you, even when they come, they wouldn't be any doctor there so they won't come, but how it's better, they are improving

I: okay I think you've mentioned some facilitating factors already, you talked about infrastructure and availability of health workers, what else do you think can be done to facilitate the integration?

R: as I said earlier, if the infrastructure is okay, then it will be left with the doctors and nurses, and the nurses are the important ones, the nurses should be compassionate enough and attend to people as a matter of urgency, when the patients come and the nurses treat them well, they will be happy to come

I: what about the community level?

R: the same thing, the nurses here go from house to house, and they treat people well so if they treat them well they will accept it, so if we get good leaders who will educate and sensitize them well they will be fine

I: okay, as a community members, what strategies do you think can be adopted to help with the integration?

R: the chiefs and opinion leaders needs to be informed, so they can announce, fix a day that will be favorable for the community members so they can he educated about the implementation of the integration, at least every quarterly the health workers can meet with the community members through the chiefs and opinion leaders so that the people can also ask questions bothering them and it will help

I: okay, thank you for your time, any other suggestions?

R: I don't know of this is relevant here but we need gutters and toilets in this community because the typhoid is too much so if they can help with our toilet facilities and gutters

I: okay, thank you for your time
