# Supplementary material for: Stakeholders perspective of integrating female genital schistosomiasis into HIV care: A qualitative study in Ghana
Source: PLoS Negl Trop Dis. 2025 Jun 2;19(6):e0012469. doi: 10.1371/journal.pntd.0012469 (PMC12169530; doi:10.1371/journal.pntd.0012469)
Supplement: Attachment — Submitted filename: Review Comments for Manuscript-Integrating FGS into HIV Care.doc [file pntd.0012469.s002.doc]

**Review comments on a manuscript titled: *“***Stakeholders Perspective of Integrating Female Genital Schistosomiasis into HIV Care: A Qualitative Study in Ghana”.

**Abstract:** The abstract is well written, with key aspects of the study captured. Authors should clearly state the design, not the approach. Merely stating that you used qualitative research methods is not sufficient.

**Introduction & background:** The authors have made great efforts to produce a good background section. Some sentences are too lengthy, they could be revised or shortened.

**Gaps and Objectives:** Gaps have been clearly identified. The objectives need to come out clearly.

**Methodology**

**Ethical considerations:** Authors have ably written this section.

**The study area** has been addressed well highlighting key issues.

**Research design:** The design chosen is clear and aligns with the study objectives.

**Sampling strategy:** Authors have presented the sampling strategy in a concise and clear manner.

**Data collection:** This section is written well and brings out the methods and tools in a simple manner.

**Data analysis:** this section was properly written. However, authors could be more detailed on the analysis methods and procedures. As it is now, readers may not understand well how the thematic content analysis was done.

**Results:** The results section is well presented with key findings highlighted. Some of the quotes do not depict the key findings. Eg on knowledge of FGS by KII R2 male, was not true. Instead, the KI participant quotes are about schistosomiasis in general, but not FGS.

**Discussion:** This section was well written and addresses all key findings.

**Conclusion and recommendations:** The authors have made good conclusions and recommendations.

**General comments:** This study makes significant contributions in the fight against both FGS and HIV/AIDs. Findings of this study could go a long way in improving management of FGS and HIV care not only in Ghana but Africa and the world.
