# Supplementary material for: Stakeholders perspective of integrating female genital schistosomiasis into HIV care: A qualitative study in Ghana
Source: PLoS Negl Trop Dis. 2025 Jun 2;19(6):e0012469. doi: 10.1371/journal.pntd.0012469 (PMC12169530; doi:10.1371/journal.pntd.0012469)
Supplement: Attachment — Submitted filename: Response to Reviewers PLOS Neglected Tropical Diseases.docx [file pntd.0012469.s005.docx]

**Comments from Editor**

- Add authors summary
- **Response:** The summary has been provided between the abstract and introduction
- Upload transcripts as supplementary file
- **Response:** This has been done and data availability statement revised to reflect the change.

**Reviewers' comments:**

Reviewer #1: Abstract: The abstract is well written, with key aspects of the study captured. Authors should clearly state the design, not the approach. Merely stating that you used qualitative research methods is not sufficient.

**Response:** We have added the study design in the abstract

Introduction & background: The authors have made great efforts to produce a good background section. Some sentences are too lengthy, they could be revised or shortened.

Gaps and Objectives: Gaps have been clearly identified. The objectives need to come out clearly.

**Response:** Thank you for your review

Methodology
Ethical considerations: Authors have ably written this section.
The study area has been addressed well highlighting key issues.
Research design: The design chosen is clear and aligns with the study objectives.

Sampling strategy: Authors have presented the sampling strategy in a concise and clear manner.

Data collection: This section is written well and brings out the methods and tools in a simple manner.
Data analysis: this section was properly written. However, authors could be more detailed on the analysis methods and procedures. As it is now, readers may not understand well how the thematic content analysis was done.

**Response:** We have expanded the description of the data analysis process and procedure

Reviewer #2: The methods applied are clear. However, the authors need to strengthen the inclusion criteria since it was purposive sampling. Note that they selected people with HIV and FGS; community health workers.

**Response:** More details have been provided and highlighted in the revised manuscript

**Results**

Reviewer #1: Results: The results section is well presented with key findings highlighted. Some of the quotes do not depict the key findings. Eg on knowledge of FGS by KII R2 male, was not true. Instead, the KI participant quotes are about schistosomiasis in general, but not FGS.

**Response:** A new illustrative quote has been provided and the old one deleted

**Response**

Discussion: This section was well written and addresses all key findings.

Reviewer #2: The results are clearly presented. Authors can consider identifying key themes from the respondents to make the paper more concise.

**Response:** Thank you for your review

**Conclusions**

Reviewer #1: Conclusion and recommendations: The authors have made good conclusions and recommendations.

Reviewer #2: The conclusions are clear however the authors need to clearly state that there was no clinical evidence between FGS and HIV but instead that the study measured the knowledge, of FGS and HIV in a select group of people.

Response: This revision has been added at the opening sentence of the conclusion

**Editorial and Data Presentation Modifications?**


Use this section for editorial suggestions as well as relatively minor modifications of existing data that would enhance clarity. If the only modifications needed are minor and/or editorial, you may wish to recommend “Minor Revision” or “Accept”.

Reviewer #1: Minor revision

Reviewer #2: Minor revision to improve clarity and ensure that the paper presents itself as measuring the knowledge of a sample of people in a country with high FGS prevalence. Statements that indicate a link between FGS and HIV should be clearly backed by evidence.

**Response:** Thank you for your review

Reviewer #1: General comments: This study makes significant contributions in the fight against both FGS and HIV/AIDs. Findings of this study could go a long way in improving management of FGS and HIV care not only in Ghana but Africa and the world.

Reviewer #2: The paper is a great contribution to a field of diseases (FGS) that are often not discussed or researched on by mainstream public health entities. It calls our attention to the need to better understand an advocate for the integration of FGS into HIV care and services. It also contributes to the global call to integrate FGS with sexual and reproductive health

**Response:** Thank you for your review
